# Characterization of an Oct1 orthologue in the channel catfish, *Ictalurus punctatus*: A negative regulator of immunoglobulin gene transcription?

**DOI:** 10.1186/1471-2199-8-8

**Published:** 2007-01-31

**Authors:** Mara L Lennard, Jun-ichi Hikima, David A Ross, Corine P Kruiswijk, Melanie R Wilson, Norman W Miller, Gregory W Warr

**Affiliations:** 1 Marine Biomedicine and Environmental Sciences Center and Department of Biochemistry and Molecular Biology, Medical University of South Carolina, Charleston, SC 29425, USA; 2 Cell Biology and Immunology Group, Wageningen Institute of Animal Sciences, Wageningen University, 6709 PG Wageningen, The Netherlands; 3 Department of Microbiology, University of Mississippi Medical Center, Jackson, MS 39216, USA

## Abstract

**Background:**

The enhancer (Eμ3') of the immunoglobulin heavy chain locus (IGH) of the channel catfish (*Ictalurus punctatus*) has been well characterized. The functional core region consists of two variant Oct transcription factor binding octamer motifs and one E-protein binding μE5 site. An orthologue to the Oct2 transcription factor has previously been cloned in catfish and is a functionally active transcription factor. This study was undertaken to clone and characterize the Oct1 transcription factor, which has also been shown to be important in driving immunoglobulin gene transcription in mammals.

**Results:**

An orthologue of Oct1, a POU family transcription factor, was cloned from a catfish macrophage cDNA library. The inferred amino acid sequence of the catfish Oct1, when aligned with other vertebrate Oct1 sequences, revealed clear conservation of structure, with the POU specific subdomain of catfish Oct1 showing 96% identity to that of mouse Oct1. Expression of Oct1 was observed in clonal T and B cell lines and in all tissues examined. Catfish Oct1, when transfected into both mammalian (mouse) and catfish B cell lines, unexpectedly failed to drive transcription from three different octamer-containing reporter constructs. These contained a trimer of octamer motifs, a fish *V*_*H *_promoter, and the core region of the catfish Eμ3'* IGH *enhancer, respectively. This failure of catfish Oct1 to drive transcription was not rescued by human BOB.1, a co-activator of Oct transcription factors that stimulates transcription driven by catfish Oct2. When co-transfected with catfish Oct2, Oct1 reduced Oct2 driven transcriptional activation. Electrophoretic mobility shift assays showed that catfish Oct1 (native or expressed *in vitro*) bound both consensus and variant octamer motifs. Putative N- and C-terminal activation domains of Oct1, when fused to a Gal4 DNA binding domain and co-transfected with Gal4-dependent reporter constructs were transcriptionally inactive, which may be due in part to a lack of residues associated with activation domain function.

**Conclusion:**

An orthologue to mammalian Oct1 has been found in the catfish. It is similar to mammalian Oct1 in structure and expression. However, these results indicate that the physiological functions of catfish Oct1 differ from those of mammalian Oct1 and include negative regulation of transcription.

## Background

The octamer motif (ATGCAAAT) and its reverse complement [1] are important *cis*-regulatory elements found in transcriptional control regions of ubiquitously expressed genes, such as histone H2B and small nuclear RNAs (U1, U2, and U6), as well as cell-type specific genes, such as the immunoglobulin (*IG*) heavy and light chain genes [2-4]. The Oct transcription factors, which bind octamer motifs, are members of the POU (Pit, Oct, Unc) family of proteins which are characterized by a bipartite DNA binding domain. The binding domain consists of two subdomains, the POU specific (POU_S_) domain and the POU homeodomain (POU_H_) that are joined by a linker region, whose flexibility allows recognition of the cognate octamer motif by binding to both faces of the DNA [5,6]. The POU proteins can be grouped, based on the structure of their POU domains, into six or more classes [7,8] POU transcription factors of Class II have been widely studied in the vertebrates, and members of this class have also been reported in ecdysozoan invertebrates such as *Drosophila *[9], and in the deuterostomate echinoderm *Strongylocentrotus purpuratus *[10], and the urochordate *Oikopleura dioica *[11]. In the vertebrates, Oct1 (which is ubiquitously expressed) and Oct2 (which is expressed predominantly in B cells and the central nervous system) are the most widely studied Class II POU transcription factors. Both Oct1 and Oct2 are essential transcription factors, with Oct1 gene knockout in mouse being embryonic lethal [12] and Oct2 knockout resulting in death shortly after birth [13].

While Oct1 is involved in directing the transcription of both Pol II and Pol III genes, Oct2 was originally thought to have a more restricted role, for example in controlling *IG *gene transcription in B cells [14,15]. However, subsequent studies [13] suggested that Oct2 is redundant, because Oct1 can also play a significant role in the transcription of mammalian *IG *genes. Depletion studies in HeLa cells and BJA-B cells indicated that both Oct1 and Oct2 could drive transcription from the *IGH *promoter in either cell line and with nearly identical binding activity [16]. In order to explain the evidence that transcription of both ubiquitously expressed and cell type restricted genes could be regulated by Oct1 or Oct2, the idea of a B cell specific co-activator arose. The discovery of BOB.1 (OBF-1 or OCA-B), a B cell specific co-activator of Oct1 and Oct2 proteins, was thought to provide the answer [17-19]. However, thorough exploration of mouse B cell development and function in the absence of Oct1[20], Oct2 [13] or BOB.1[21], or a combination of Oct2 and BOB.1[22], showed surprisingly little effect on *IGHM *gene transcription. Some effects were noticed in the production of secondary isotypes (IGHG and IGHA) and in the formation of germinal centers. Thus, although the details of the mechanisms controlling the expression of *IG *genes in mammalian B-cells remain unclear, neither Oct1 nor Oct2 are essential transcription factors for this function, and the potential for comparative studies to shed light on the essential and evolutionarily-conserved aspects of IG gene transcription is considerable.

The channel catfish provides a system for studying the transcriptional control of teleost *IG *genes, particularly with respect to the heavy chain (*IGH*) locus where octamer motifs and octamer-binding transcription factors have been shown to play important roles [23]. Catfish express only two classes of immunoglobulin (IGHM and IGHD) and class switching by chromosomal recombination is absent [24-26]. A single enhancer, Eμ3', has been found associated with the expressed *IGHM1 *and *IGHD1 *genes of the catfish [27,28] and is situated immediately 3' of the *IGHM1 *gene. The core region of this enhancer consists of two variant (but fully functional) octamer motifs and a μE5 site. It has been previously established that octamer-binding transcription factors are important in driving transcription from the catfish Eμ3' enhancer [23]. This situation in the catfish differs from what is seen with the mammalian *IGH *locus, where the Eμ enhancer is located 5' of the constant region gene cluster and contains a single octamer motif, which is not within the core enhancer [29,30]. Catfish Oct2 has been cloned and shown to be expressed as two predominant isoforms, Oct2α and β, derived from a single gene by alternative RNA processing. Both catfish Oct2 isoforms are transcriptionally active [31], although the β isoform is a more potent transactivator than Oct2α due to differences in the C-terminal proline, serine, threonine rich activation domains of the two isoforms [32]. While the role of catfish Oct2 in transcriptional control of the *IGH *locus of the channel catfish is well understood, the functional significance of other Oct transcription factors has not yet been determined. The study reported here was undertaken to clone the catfish orthologue of Oct1, and to characterize its expression and function in driving transcription of the *IGH *locus in B cells.

## Results

### Identification of an Oct1 orthologue in channel catfish

**Figure 1:**
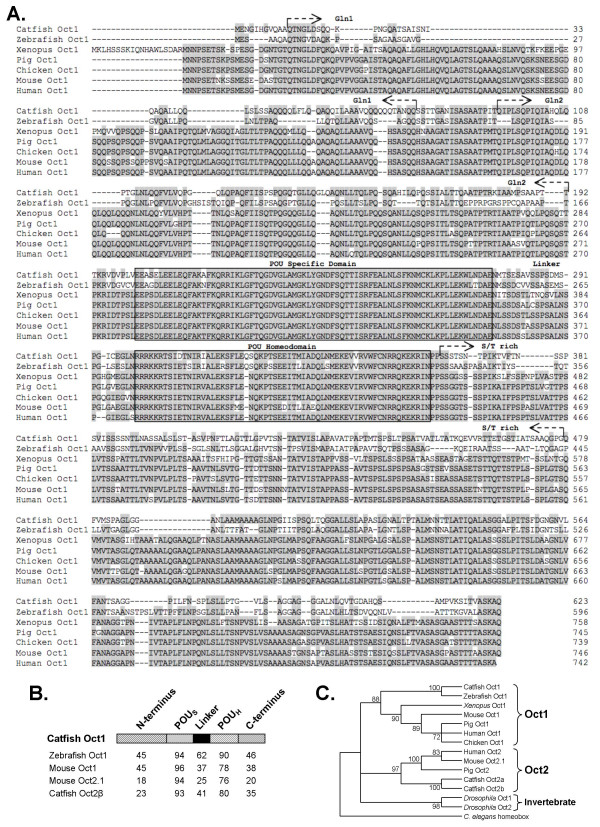
**An Oct1 orthologue in catfish**. A. Megalin (DNAStar) amino acid alignment of Oct1 proteins. Conserved residues are shaded by grey boxes. The boxed portion indicates the bipartite POU DNA binding domain. Glutamine rich regions are denoted by arrows and text Gln1 or Gln2. Serine and thrionine rich residues are denoted by arrows and S/T rich. The linker region is also designated by text. Gaps in the sequence alignment are indicated by a dash. B. Schematic comparing the domain structure of Oct transcription factors. N- and C- terminal domains are shown as checkered boxes, POU domains are shown hatched with diagonal lines, and the linker region is shown as a black box. The percent identity values (derived using the Megalign program, DNAStar) are based on the amino acid sequences and are shown below the schematic. C. Neighbor joining phylogenetic tree [49] of Oct1 and Oct2 transcription factor sequences (see Methods). The *C. elegans *homeobox gene was used as the outgroup. The three branches for Oct1, Oct2 and the invertebrate Oct sequences are indicated by brackets, and the bootstrap value for each node is shown.

A cDNA library from a catfish monocyte cell line (42TA) was screened with a ^32^P-labeled probe for the POU domain of catfish Oct2 (see Methods), and a full-length clone with putative homology to vertebrate Oct1 was identified. An amino acid sequence alignment of Oct1 genes can be found in Fig 1A. From the alignment a schematic of the inferred structure of catfish Oct1 was made and is shown in Fig 1B. The amino acid alignment confirmed the high level of conservation within the POU specific domain and the POU homeodomain (96% and 78% with the homologous mouse Oct1 domains, respectively). The putative activation domains N- and C-terminal to the POU domain also showed substantial sequence identity, ranging between 36% and 46% in comparison with the mouse and zebrafish Oct1 activation domains. A more comprehensive analysis of the evolutionary relationships of catfish Oct1 is shown in the phylogenetic tree of Fig 1C. This demonstrates that the separation into Oct1 and Oct2 occurred early in vertebrate evolution, and that the invertebrate Oct genes form an outgroup and cannot be classified clearly as either Oct1 or Oct2 othologues [33]. Although isoforms of catfish Oct2 have easily been identified [31], analysis by RT-PCR and RACE-PCR of cDNA from catfish B cell and T cell lines (see Methods) did not identify any sequences of catfish Oct1 other than the one originally isolated (Fig 1).

### Catfish Oct1 is widely expressed

**Figure 2:**
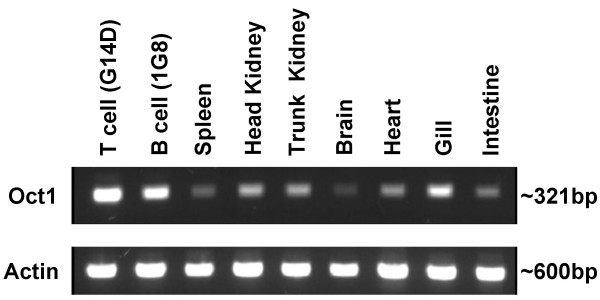
**Catfish Oct1 is widely expressed**. RNA was isolated from the catfish T cell line (G14D) and B cell line (1G8) as well as catfish tissues (spleen, head kidney, trunk kidney, brain, heart, gill, and intestine) and subjected to RT-PCR analysis to detect Oct1 (top panel), as described in the Methods section. RT-PCR detection of the catfish actin transcript is shown in the bottom panel.

Ubiquitous expression in cells and tissues is one of the key features that distinguishes mammalian Oct1 from the tissue-restricted Oct2. Therefore, the expression of catfish Oct1 in clonal T and B cell lines and in a variety of tissues was analyzed by RT-PCR (Fig 2). The primers used in the RT- PCR analysis were designed within the C-terminus of the catfish Oct1 to distinguish the Oct1 gene from the two isoforms of catfish Oct2. The primer sequences were not found within the sequence for either isoform of catfish Oct2 and NCBI blast search returned matches only to the catfish Oct1 sequence. The cloned, catfish B and T cell lines both expressed Oct1, as did all of the tissues examined, including spleen, head and trunk kidney, brain, heart, gill and intestine. This pattern of widespread expression is consistent with its mammalian counterparts, and with the expectation that it would play a significant role in gene regulation. Thus, the ability of catfish Oct1 to drive expression from a variety of octamer-containing reporter constructs was tested.

### Transcriptional Activity of catfish Oct1

**Figure 3:**
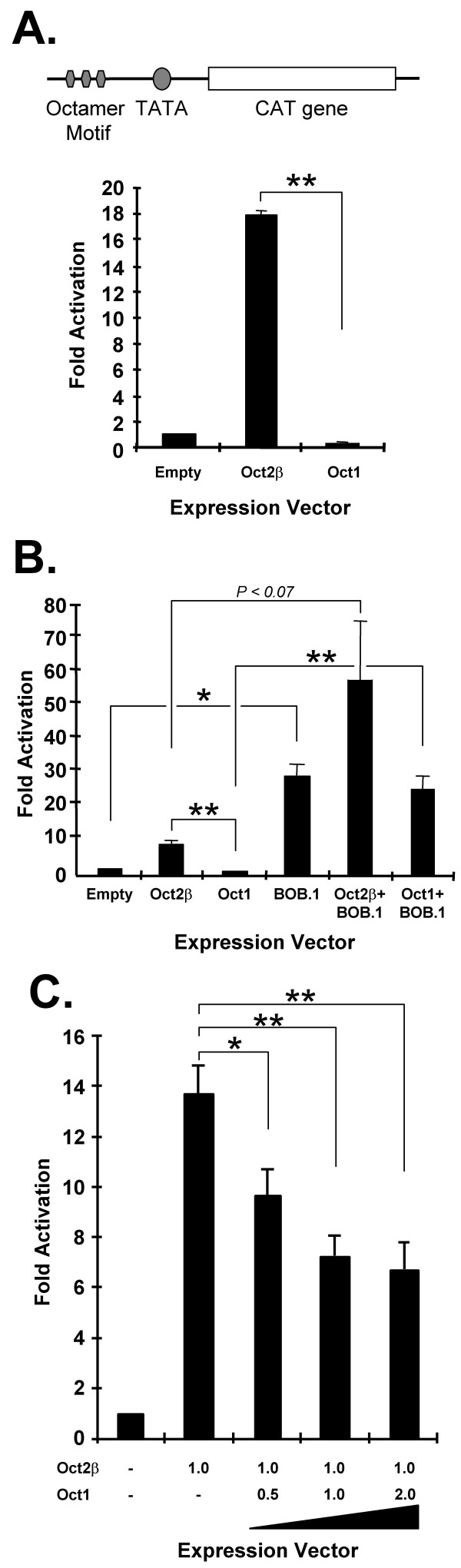
**Catfish Oct1 fails to drive transcription from an octamer-dependent reporter construct**. A schematic representation of the reporter construct (pO3-Δ56-CAT [31]) containing an octamer trimer is shown in A. The octamer motifs are shown upstream of the TATA box (from the minimal *c-fos *promoter) and the CAT reporter gene is shown as an open box. Transcriptional activation (as fold increase) was measured following co-transfection into the catfish B cell line (1G8) of the reporter construct and an Oct expression construct. A. Transcriptional activation driven by catfish Oct2β (pRc/CMV/Oct2β) or catfish Oct1 (pRc/CMV/Oct1). B. Transcriptional activation driven by catfish Oct2β (pRc/CMV/Oct2β), catfish Oct1 (pRc/CMV/Oct1) and human BOB.1 (pRc/CMV/hBOB.1), or by a co-transfection of catfish Oct2β with human BOB.1, or catfish Oct1 with human BOB.1. C. Negative transcriptional regulation by catfish Oct1. One pM of catfish Oct2β expression construct was co-transfected with increasing amounts of catfish Oct1 (0.5, 1.0, 2.0 pM respectively) and the transcriptional activation of the reporter construct measured. Values for five replicate transfections in A and four replicate transfections in B and C are shown as mean ± SEM. Statistical significance was calculated by the Student T test assuming unequal variances. One asterisk indicates a p value of less than .05 and two asterisks indicates a p value of less than .01.

**Figure 4:**
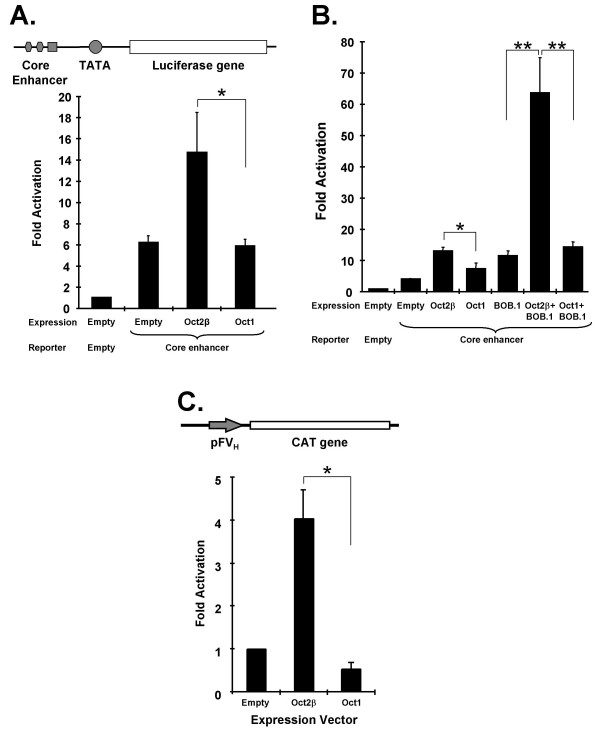
**Catfish Oct1 fails to drive transcription from physiologically relevant reporter constructs**. A. Schematic diagram of the reporter construct pGL3/Δ56/R#2 [50]. This contains the core of the catfish enhancer in which there are 2 octamer motifs (ovals) and a μE5 site (square) upstream of a minimal TATA box (circle) followed by the luciferase gene (open rectangle). This reporter construct was used in the experiments shown in panels A&B. A. Transcriptional activation driven from the core enhancer by catfish Oct2β (pRc/CMV/Oct2β) or catfish Oct1 (pRc/CMV/Oct1) in the mouse B cell line (J558L). B. Transcriptional activation in the catfish B cell line 1G8 driven from the core enhancer construct by catfish Oct2β (pRc/CMV/Oct2β), catfish Oct1 (pRc/CMV/Oct1), human BOB.1 (pRc/CMV/hBOB.1), or by a co-transfection of catfish Oct2β with human BOB.1, or catfish Oct1 with human BOB.1. C. (Top) Schematic diagram of the reporter construct (pFVH-CAT) that contains a full fish *V*_*H *_promoter (arrow) followed by the CAT gene [27]. C. (Bottom) Transcriptional activation driven from the *V*_*H *_promoter (pFVH-CAT) by catfish Oct2β (pRc/CMV/Oct2β) or catfish Oct1 (pRc/CMV/Oct1) co-transfected with the reporter construct into catfish 1G8 B cells. Values are presented as mean ± SD for 12 replicates (A), 9 replicates (B), and 4 replicates (C). Statistical significance was calculated by the Student T test assuming unequal variances. One asterisk indicates a p value of less than .05 and two asterisks indicate a p value of less than .01.

An Oct1 expression vector was co-transfected, with several different reporter constructs, all of which contained octamer motifs, into catfish B cell lines. Oct1 was, surprisingly, unable to drive transcription from an octamer-dependent reporter that contains a trimer of octamer motifs upstream of a minimal *c-fos *promoter, and which is responsive to catfish Oct2 (Fig 3A). Whilst the transcription driven by the transfected Oct1 was actually found to be significantly suppressive (p < .01) from the empty vector control, catfish Oct2, as predicted, induced transcription strongly. We considered the possibility that Oct1 requires a co-activator to function, and thus examined its ability to act in concert with human BOB.1, a co-activator of Oct transcription factors. BOB.1 has previously been shown to activate transcription synergistically with catfish Oct2 [34] but as shown in Fig 3B, human BOB.1 was unable to act synergistically with catfish Oct1. This was not due to a lack of function of BOB.1, as it enhanced transcription (at a statistically significant level) from the octamer-dependent reporter when transfected alone into catfish B cells, presumably through interaction with the endogenous Oct transcription factors. The interaction of human BOB.1 with catfish Oct2 gave transcription that was increased above the level seen with either BOB.1 or Oct2 transfected alone (Fig 3B). Whilst this interactive effect of BOB.1 on Oct2-driven transcription did not reach statistical significance (p < 0.07), in a second series of experiments (described below) this effect was confirmed (see Fig 4B). The possibility that catfish Oct1 and Oct2 might act synergistically was tested in a series of co-transfection experiments. The results (Fig 3C) showed that the transcriptional activation produced by catfish Oct2 was in fact reduced (in a statistically significant manner) with increasing amounts of co-transfected Oct1. In order to test if the inability of catfish Oct1 to drive transcription was reporter-dependent, its activity was tested on two other physiologically-relevant reporter constructs. The ability of catfish Oct1 to drive transcription from a reporter construct containing a minimal promoter and the core region of the enhancer (Eμ3') of the catfish *IGH *locus was tested in both mouse J558L plasmacytoma cells and in the catfish B cell line 1G8. The results show that this reporter, while responsive to catfish Oct2, was unresponsive to Oct1 both in mouse (Fig 4A) and catfish (Fig 4B) cells. The activation by catfish Oct2 was statistically significant (when compared to the reporter construct alone) activation by catfish Oct1 was not. When catfish Oct1 and human BOB.1 were co-transfected, the level of transcriptional activation was no higher than that seen with BOB.1 alone (Fig 4B), confirming the inability of catfish Oct1 to interact positively with this co-activator. Co-transfection of human BOB.1 with catfish Oct2 enhanced transcription to a statistically significant extent, supporting the effect suggested by the results shown in Fig 3B. The activity of catfish Oct1 was then tested with a third octamer-containing reporter construct, in which expression is driven from a fish *V*_*H *_gene promoter that contains a single octamer motif (Fig 4C). While this construct was highly responsive to Oct2, it failed to respond to co-transfected Oct1 (Fig 4C) and these results were also found to be statistically significant. Given these unexpected results, the possibilities were next considered that catfish Oct1 was either a) unable to bind to an octamer motif or b) if it could bind to an octamer motif, it lacked functional activation domains.

### *In vitro *expressed catfish Oct1 binds the octamer motif

**Figure 5:**
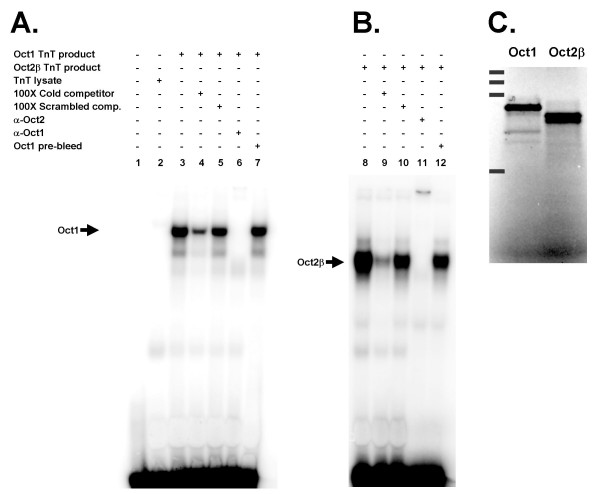
***In vitro *transcribed Oct transcription factors bind the octamer motif**. A. EMSA demonstrating the binding of catfish Oct1 to the first octamer motif within the core enhancer. Lane 1, free probe. Lane 2, TNT reaction without template DNA (control). Lane 3, catfish Oct1 TNT product. Lane 4, catfish Oct1 TNT product + cold competitor. Lane 5, catfish Oct1 TNT product + scrambled competitor. Lane 6, catfish Oct1 TNT product + anti-Oct1 rabbit serum. Lane 7, catfish Oct1 TNT product + NRS (anti-Oct1 rabbit pre-bleed serum). Arrow depicts the Oct1 shift. B. EMSA demonstrating the binding of catfish Oct2β to the first octamer motif within the core enhancer. Lane 1, catfish Oct2β TNT product. Lane 2, catfish Oct2β TNT product + cold competitor. Lane 3, catfish Oct2β TNT product + scrambled competitor. Lane 4, catfish Oct2β TNT product + anti-Oct2β rabbit serum. Lane 5, catfish Oct2β TNT product + NRS (anti-Oct1 rabbit pre-bleed serum. C. Detection of ^35^S-labeled TNT transcribed and translated Oct1 and Oct2β by SDS-PAGE and phosphor imaging. Solid black bands on the left hand side represent the kaleidoscope protein marker (Bio-Rad, Hercules, CA.), 222kDa, 129kDa, 83kDa, 43kDa (from top to bottom).

In order to test the ability of catfish Oct1 to bind to the octamer motif, catfish Oct2β and catfish Oct1 proteins were produced by *in vitro *transcription and translation and tested by EMSA for their ability to bind the native octamer motif (ATGtAAAT) found within the catfish core enhancer (Fig 5). The first two lanes of the gel shift show the free probe (Fig 5A, Lane 1) and the TNT lysate (Fig 5A, Lane 2) without the addition of any DNA as controls. These results show that Oct1 bound to the octamer motif (Fig 5A, Lane 3), and that the binding was specifically inhibited by an excess of unlabeled octamer motif (Fig 5A, Lane 4) but not by an excess of the octamer sequence that had been scrambled (Fig 5A, Lane 5). Addition of the antibody to catfish Oct1 eliminated the shifted band (Fig 5A, Lane 6), indicating that the observed shift was Oct1-induced. The control (preimmunization bleed) serum did not affect the observed shift (Fig 5A, Lane 7). The ability of catfish Oct2β to bind to the native octamer motif was confirmed by the results (shown in Fig 5B) which are similar to those observed with catfish Oct1. The *in vitro *synthesized proteins were checked for size by radiolabeling with ^35^S methionine and analyzed on an SDS PAGE gel (figure 5C). The sizes of the protein bands produced for catfish Oct1 and Oct2β were consistent with the expected sizes of the proteins, 65kDa for catfish Oct1 and 52kDa for catfish Oct2β.

### Octamer-binding transcription factors of catfish B cells

**Figure 6:**
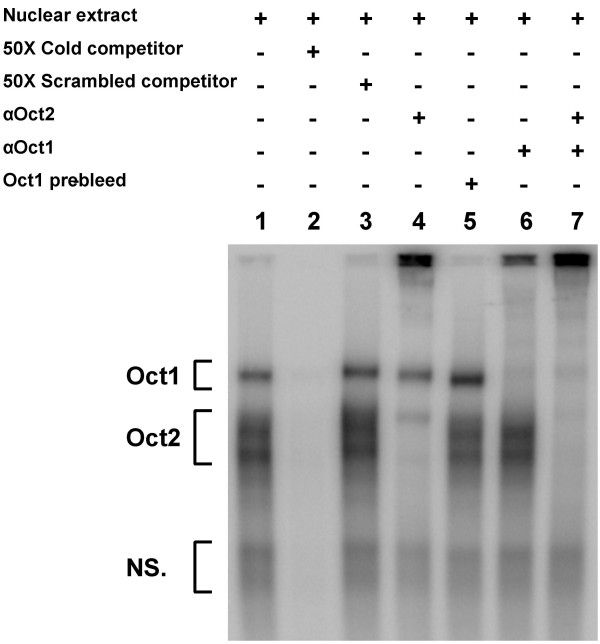
**Endogenous Oct transcription factors bind the octamer motif**. EMSA with nuclear extracts of the catfish B cell line 1G8. Lane 1, nuclear extract. Lane 2, nuclear extract + cold competitor. Lane 3, nuclear extract + scrambled competitor. Lane 4, nuclear extract + anti-Oct2 rabbit serum. Lane 5, nuclear extract + NRS (anti-Oct1 rabbit pre-bleed serum. Lane 6, nuclear extract + anti-Oct1 rabbit serum. Lane 7, nuclear extract + anti-Oct1 rabbit serum + anti-Oct2 rabbit serum. Oct1 and Oct2 shifts are designated by brackets. A lower bracket designates non specific binding labeled NS.

The ability of endogenous transcription factors expressed in catfish B cells to bind the octamer motif was also tested by EMSA. Nuclear extracts from the catfish 1G8 B cell line generated complex patterns of shifted bands when tested with a consensus octamer motif (Fig 6, Lane 1). However, all the shifted bands were abolished by an excess of unlabeled octamer motif (Fig 6, Lane 2), but not by the addition of a scrambled octamer motif (Fig 6, Lane 3) and interpretation of the banding pattern was made possible using antisera specific to Oct1 and to Oct2. The uppermost shifted band is clearly attributable to Oct1, as seen from the supershift of this band in the presence of antibody to Oct1 (Fig 6, Lanes 6, 7). Below this Oct1 shift is an envelope of strong bands attributable to Oct2, as evidenced by their supershift with antibody to Oct2 (Fig 6, Lanes 4, 7). Antibody to Oct2 failed to supershift a faint band within this lower Oct2 envelope, but the combination of antibody to Oct1 and to Oct2 supershifted all bands in the upper region of the gel (Fig 6, Lane 7). The nature of the lower, diffuse bands observed in the EMSA is not known, but they were not supershifted by the antibodies to either Oct1 or Oct2 (Fig 6, Lanes 4–7).

### Catfish Oct1 lacks functional activation domains

**Figure 7:**
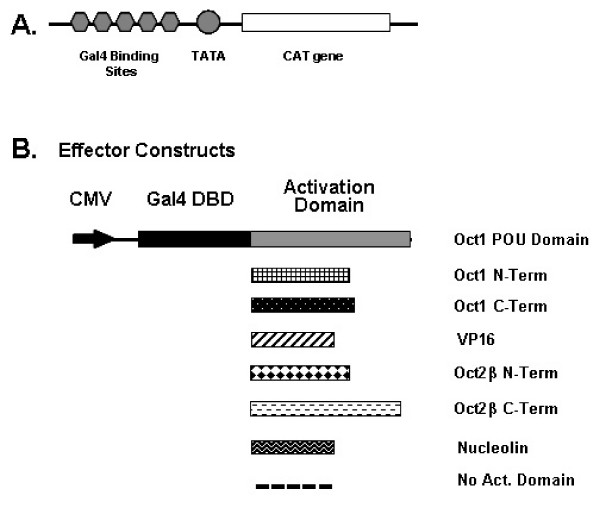
**Schematic of Gal4 fusion proteins**. A. Depicts the pG5/CAT reporter construct. Gal4 binding sites (grey hexagons), TATA promoter (grey circle) and the CAT gene (open rectangle). A schematic of the effector constructs used in B. CMV promoter (black arrow), Gal4 DNA binding domain (black rectangle), Oct1 POU domain (grey rectangle), Oct1 N-terminus (plaid rectangle), Oct1 C-terminus (black with white dotted rectangle), VP16 (diagonal stripped rectangle), Oct2β N-terminus (black diamond rectangle), Oct2β C-terminus (white with black dotted rectangle), Nucleolin (tire track rectangle), empty activation domain (dotted line).

The above results clearly demonstrate that catfish Oct1 is capable of binding to the octamer motif. Thus, in order to address the failure of catfish Oct1 to activate transcription, the function of the N- and C-terminal putative activation domains was tested. Expression constructs containing the Gal4 DNA binding domain fused to Oct1 and Oct2 putative activation domains were generated, and tested by co-transfection with a reporter constructs containing Gal4 binding sites in the promoter region (Fig 7A, B). These constructs were co-transfected into catfish B cells (the 1G8 cell line) and their ability to drive transcription from the reporter construct is shown in Table 1. The negative controls (Gal4 binding domain fused to nucleolin or to the Oct1 POU domain) were inactive or suppressive, whereas the positive control (Gal4 binding domain fused to the VP16 activation domain) showed strong statistically significant activity. The C-terminal domain of the catfish Oct2β isoform was also strongly active and statistically significant, giving a greater than 300-fold stimulation of expression. There was low activation (3-fold) seen with the N-terminus of catfish Oct2β; this level of activity is consistent with what has previously been reported and nearly statistically significant with a p-value of .057[32]. However, in the case of catfish Oct1, both the C-terminal and N-terminal domains are lacking in substantial activation ability (Table 1). While there was a slight activation (2-fold) with the C terminal domain, this activation was negligible and not statistically significant when compared to that of the catfish Oct2β C-terminus. In the case of the N-terminal domain of Oct1, it suppressed rather than enhanced transcription, this suppression was also seen to be significant from the DNA binding domain alone. Examination of the Oct1 sequence alignment in Figure 1 shows the lack of several gln residues known to be important in the N-terminal activation domain of mammalian Oct1[14,37,38]. Within the Gln1 region (Fig. 1A), which is located in the human Oct1 sequence from amino acid 22–145, 52% of the gln residues found in the human sequence are missing in catfish Oct1 and 68% are missing in the zebrafish Oct1 sequence. In the Gln2 region (amino acid 163–268 in human Oct1), 42% of the gln residues are missing in the catfish and 58% are missing in the zebrafish Oct1. The absence of these gln residues suggests that teleost Oct1 may not possess the capability to act as a transcriptional regulator. Further examination of the ser/thr rich region [14,37] located from amino acid 441–560 in human Oct1 indicates a similar pattern, i.e. 67% and 69% of ser and thr residues present in the human sequence are absent in the homologous regions of catfish and zebrafish Oct1, respectively.

## Discussion

The conclusion that the cloned catfish molecule identified in this study is a homologue of Oct1 is supported by 4 observations. First, it shows well-conserved sequence similarity to other vertebrate Oct1 (especially in the POU domain); secondly, in phylogenetic analyses it clusters to the same tree branch as other vertebrate Oct1 sequences; thirdly, it binds the octamer DNA sequence motif; and fourthly, it shows the widespread pattern of expression characteristic of vertebrate Oct1. In human and mouse several isoforms of Oct1 have been isolated, arising from alternative splicing involving the 21 expressed exons of this gene, [35]. In contrast, examination of catfish macrophage and B cell lines by RT-PCR and RACE failed to identify any alternatively spliced isoforms of catfish Oct1. However, EMSA analysis of endogenous octamer-binding transcription factors (Fig 6) suggested the possibility that an additional minor isoform of Oct1 might be expressed in catfish B cells. It is possible a) that an Oct1 isoform may be expressed at such a low level that the technique used may have failed to detect its presence or b) the primers designed for this study may have been placed in exons not included in the transcript. Analysis of the teleost fish genomes currently being sequenced revealed orthologues of the Oct1 in the zebrafish *Danio rerio*, (ENSDARG00000007996), the puffer fishes *Takifugu rubripes *(NEWSINFRUG00000159819) and *Tetraodon nigroviridis *(GSTENG00025967001), and the three-spined stickleback *Gasterosteus aculeatus *(ENSGACG00000003150). Interestingly, in only one of these species, the stickleback is Oct1 reported to have more than one transcript. Thus, despite the whole genome duplication that occurred in teleost fishes, to date no evidence for more than a single Oct1 gene has been found. While it is likely that only one Oct1 gene exists in the teleost fishes (including catfish) this question will not be resolved until the sequencing and assembly of the teleost genome sequences is completed

Although orthologues of Oct1 are present in the genomes of four other teleost fish this study represents the first examination of the function of a teleost Oct1 orthologue. Contrary to our expectations, catfish Oct1 was clearly unable to activate transcription, even though it binds to the octamer motif. This inability to drive transcription was seen using three different octamer-containing reporter constructs transfected into both mouse and catfish B cells. Not only did the catfish Oct1 fail to activate transcription from a reporter construct with a synthetic trimer of octamer motifs in the promoter region, but it also failed to activate transcription from two constructs driven by physiologically-relevant octamer-containing elements; in one case a complete fish *V*_*H *_promoter [36], and in the other case the core region of the Eμ3' enhancer of the channel catfish *IGH *locus [23]. In all cases, while the catfish Oct2 drove transcription from the reporter constructs to a statistically significant level, catfish Oct1 did not. Binding of both native and *in vitro *expressed catfish Oct1 was shown with both the consensus octamer motif (ATGCAAAT) or the variant octamer motif (ATGtAAAT) located within the core region of the catfish enhancer Eμ3'. To dissect the molecular basis for the lack of transcriptional activation of catfish Oct1, a Gal4 fusion study of the putative activation domains was undertaken. The results of this study clearly demonstrate the lack of activity of the putative activation domains in both the N- and C-terminus of the catfish Oct1 possibly associated with the relative lack of residues known to be associated with activation domain function, i.e. gln residues in the N-terminal domain and ser and thr residues in the C-terminal domain of catfish Oct 1. Co-transfection studies involving catfish Oct1 and Oct2 suggested that catfish Oct1 could be a negative regulator of transcription, in that it significantly inhibited activation driven by Oct2. The mammalian co-activator BOB.1 has been shown to act synergistically with both mammalian Oct1 and Oct2 to drive transcription [19], and the possibility was considered that this co-activator might rescue the activation function of catfish Oct1. However, while co-transfection studies confirmed that human BOB.1 is capable of significantly enhancing transcriptional activation driven by catfish Oct2β (Figures 3 and 4 and [34]), BOB.1 did not confer transcriptional activation ability on catfish Oct1. The failure of BOB.1 and catfish Oct1 to function in a synergistic manner could result from impaired physical interaction between BOB.1 and catfish Oct1. However, the BOB.1/Oct interaction is through the Oct POU domain [39] which is the most highly conserved region of the catfish Oct1 molecule (Fig 1). All of the residues in the mammalian Oct1 POU domains that are known to interact with BOB.1, including Leu6, Glu7, Leu9, Glu10, Leu53, Asn54, Phe57, Met60 within the POU_S _domain and Lys55, Arg58, Ile59 within the POU_H _domain [39] are conserved in catfish Oct1. This suggests the possibility that catfish Oct1 might function in concert with endogenous co-activators, such as a catfish homologue of BOB.1. However, if this were the case, transcriptional activation by Oct1 expressing constructs transfected into catfish B cells would have been predicted. These results suggest, collectively, a potential mechanism of transcriptional control in the catfish *IGH *locus whereby Oct1, as a negative regulator of transcription and Oct2, as an activator of transcription compete for binding to the octamer motifs found within the intronic enhancer (Eμ3') and the *V*_*H *_promoters [27,40].

Mammalian Oct1 is involved in driving transcription from both Pol II and Pol III promoters and also has a role in recruiting basal transcription factors such as SNAPc [41]. In addition, mammalian Oct1 demonstrates promoter-selective activation domains and its ability to drive mRNA transcription can be weaker than that of Oct2 [14,42,43]. While mammalian Oct1 has not been reported to act as a negative regulator of *IG *gene transcription, its very diverse functions in regulating the expression of other genes include both positive and negative effects. In the case of the Gonadotropin-Releasing Hormone Receptor (*GnRHR*) gene, Oct1 has been shown to be capable of both activation and repression [44]. The POU domain of Oct1 interacts with the silencing mediator for retinoid and thyroid receptors (SMRT), recruiting histone deacetylases to the promoter and leading to transcriptional repression [45]. Two more recent studies have indicated additional roles for Oct1. Mouse embryo fibroblast (MEF) cell lines depleted of Oct1 showed an arrest in Herpes simplex virus replication, due to a role for Oct1 in aiding viral assembly as well as a likely secondary role affecting viral DNA replication [46]. A recent study of gene expression has also indicated that Oct1 can act as a stress sensor. Mouse fibroblasts deficient in Oct1 showed altered expression in a variety of genes known to be involved in the response to oxidative and metabolical stress [47]. In both of these cases the DNA binding ability of Oct1 is critical.

## Conclusion

The study reported here is the first demonstration that an Oct1 protein has an inhibitory effect on the transcriptional regulatory elements of *IG *genes. However, given that mammalian Oct1 has multiple and diverse roles, it is possible that Oct1 in teleosts has significantly different roles from those seen in mammals, relating not only to the transcription of genes but to the response to cellular stress and viral infection.

## Methods

### Cloning and Sequence Determination of Catfish Oct1

A cDNA library was prepared in λ ZAPII (Stratagene, La Jolla, CA) from the 42TA catfish monocyte/macrophage line [48]. Approximately 2.5 × 10^5 ^pfu were screened at high stringency with a ^32^P-labeled probe to the POU domain of catfish Oct2 [31]. Two positive clones were obtained and plaque purified. One of these contained a full-length Oct1, and its sequence (GenBank Acc#AJ000267) was determined from both strands by primer-extension sequencing (Biomolecular Resource Laboratory of the Medical University of South Carolina). Isoforms of Oct1 were sought by 3'-RACE using forward primers at positions 1–21 (G-1594); 540–569(G-2338); 547–567 (G-1595); 943–966 (G-2335); 1063–1083 (G-1596); 1836–1856 (G-1597), by 5'-RACE using reverse primers at positions 963–986 (G-2336); 1169–1195 (G-2337) and by RT-PCR using these primers in appropriate combination with reverse downstream primers at positions 615–635 (G-1598); 1206–1226 (G-1599) and 1836–1856 (G-1600). An additional reverse primer in the 3'UTR of the Oct1 clone, G-1593 (5'-AATAAAGTCTAAAGAGCGAGG-3') was also used.

### Phylogenetic Analysis

Sequence alignments were carried out using Clustal V in the Megalign suite of programs (DNA Star, Madison, WI) with PAM 250, gap length penalty of 10 and gap penalty of 10. Phylogenetic and molecular evolutionary analyses were conducted using MEGA version 2.1 [49]. The neighbor joining (NJ) phylogenetic tree of Oct1 full-length amino acid sequences was generated with 1000 bootstrap replications. The following amino acid sequences (accession numbers) were used to generate the NJ tree. Catfish Oct2α (Y12651), Catfish Oct2β (Y12652), Catfish Oct1 (AJ00267), Human Oct2 (X13810), Human Oct1 (X13403), Pig Oct2 (Q29013), Pig Oct1 (L38524), Mouse Oct2.1 (X57936), Mouse Oct1 (X68363), Chicken Oct1 (M29972), *Xenopus *Oct1 (X57165), Zebrafish Oct1 (NM131438), *Drosophila *Oct2 (S80561), *Drosophila *Oct1 (S80559), and the *Caenorhabditis. elegans *homeobox protein (Z79757).

### RT-PCR

Total RNA was isolated from the head kidney, trunk kidney, spleen, brain, heart, gill and intestine from catfish and from the B lymphoblastoid cell line (1G8) and the T-cell lines (G14D) of the catfish using Trizol (Invitrogen Life Technologies, San Diego, CA). The RNA was then treated with DNase I by addition to RNAeasy mini-kit columns (Qiagen, Valencia, CA). After elution from the columns 0.5 μL of ribonuclease inhibitor, 40u/μl (RNasin, Promega, Madison, WI), was added to the RNA. First strand synthesis was then completed using 4 μL of total RNA, an oligo dT primer, and PowerScript Reverse Transcriptase (BD Biosciences Clontech, Palo Alto, CA). The concentration of cDNA was measured and diluted to 1 μg/μL. A final concentration of 4 ng/μL was used in the PCR reaction with Ex Taq polymerase (Takara, Shiga, Japan). The PCR program was performed for 30 cycles and used the following profile: 30 sec at 95°C; 30 sec at 55°C; 2 min at 72°C. The following primers, located within the C-terminus of the Oct1 gene, were used in the RT-PCR analysis.

Primers:

For Oct1

G-1392 – 5' CGT CGC TTA CTC CCT CTG CTA C 3'

G-2890 – 5' GAT GCA AGA GCC TGA ATA GTG 3'

For Actin

G-1480 – 5' CCC ACA CTG TGC CCA TCT ATG A 3'

G-1481 – 5' GAC AGG GAG CCC AGG ATT GAG 3'

### Reporter Constructs and Expression Vectors

Three octamer dependent reporter constructs were used. 1) a luciferase expressing construct, pGL3/Δ56/R#2, which contains the core region of the catfish Eμ3' enhancer upstream of the minimal *c-fos *promoter (described in [50], 2) a chloramphenicol acetyltransferase expressing reporter, pO3-Δ56-CAT, with a trimer of octamer motifs (ATGCAAAT) upstream of the minimal *c-fos *promoter [31], and 3) a chloramphenicol acetyltransferase expressing reporter, pFVH-CAT, with a complete goldfish VH gene promoter [36]. The catfish Oct2β expression vector has been previously described [31]. The catfish Oct1 was directionally cloned (using *Not*I and *Apa*I sites) into the pRc/CMV expression vector. Coding sequences were amplified by PCR (forward primer G-2203 – 5' AAG GAA AAA AGC GGC CGC **GGC ACC **ATG GAG AAT GGA ATA CAT GGA GTC C 3', reverse primer G-2179 – 5' TTT GGG CCC TCA TTG CGC CTT GGA GGC CTC 3'). The restriction sites are underlined and a Kozak consensus sequence is shown in bold in the sense primer. The human BOB.1 vector, expressed from pRc/CMV, was a generous gift from P. Matthias (Friedrich Miescher Institute for Biomedical Research, Novartis Forschungsstiftung, Basel, Switzerland).

For the Gal4 fusion study the reporter construct pG5/CAT was used [32]. This construct contains 5 Gal4 binding sites, located upstream of a minimal TATA box promoter and the CAT reporter gene. The expression constructs for the fusion proteins containing the Gal4 DNA binding domain (DBD) alone or with VP16; nucleolin; catfish Oct2β N-terminus; and catfish Oct2β C-terminus have previously been described [32]. The Gal4DBD-Oct1 domain fusion expression constructs were produced by PCR and were directionally cloned into the Gal4DBD expression construct with *XbaI *and *BamHI *restriction enzyme sites. An internal stop codon was also included prior to the *BamHI *site. All clones were checked by sequencing before use to ensure the absence of PCR-induced mutations.

Primers:

Oct1 N-terminus

G – 2977 – 5' TTT TCT AGA GAG AAT GGA ATA CAT GGA GTC CAA 3'

G – 2978 – 5' TTT GGA TCC CTA TAG AGG AAC ATC CAC CCG CTT 3'

Oct1 POU domain

G – 2979 – 5' TTT TCT AGA GTG GAG GAG GCC AGT GAA CTG 3'

G – 2980 – 5' TTT GGA TCC CTA GTT GAT CCT CTT CTC CTT CTG 3'

Oct1 C-terminus

G – 2981 – 5' TTT TCT AGA AAC CCC CCG AGC AGC AGC ACA 3'

G – 2982 – 5' TTT GGA TCC CTA TCA TTG CGC CTT GGA GGC CAC 3'

### Cell Lines and DNA transfection

The catfish B lymphoblastoid cell line 1G8 and T cell line G14D, and the mouse plasmacytoma cell line J558L were maintained as previously described [50]. Transfections were performed with an Electro Cell Manipulator 600 (BTX, San Diego, Ca) and 2-mm gap cuvettes (BTX) were used. All cells were harvested during the logarithmic growth phase and washed once in serum free media (45% AIMV, 45% L15 and 10% deionized water for catfish cells and RPMI-1640 for mouse cells) before resuspension in serum free media prior to transfection. Immediately before transfection 180 μL of cells (8 × 10^6 ^cells of 1G8 and 4 × 10^6 ^cells of J558L) were mixed with 20 μL of prepared DNA. Reporter construct (2.4 pM of pGL3/Δ56/R#2), (3.2 pM of pFVH-CAT), (3.3 pM of pO3-Δ56-CAT) and empty, Oct1, Oct2 or BOB.1 expression vectors (1.6 pM) were transfected into the cells. For competition studies 1 pM of Oct2 construct was transfected, and increasing amounts of the Oct1 construct were added; 0.5 pM, 1 pM, and 2 pM. For the Gal4 studies 3.95 pM of the pG5/CAT reporter construct was used. For the expression constructs, 1.108 pM of Gal4-DBD alone, Gal4DBD bound to nucleolin, VP16, Oct2β N-Term, Oct2β C-term, Oct1 POU domain, Oct1 N-term, Oct1 C-term was transfected into the catfish 1G8 cell line. In all experiments 1 μg of a *Renilla *luciferase construct with a CMV promoter (pRL/CMV; Promega) was used as the transfection control. Optimal transfection conditions were used as previously described [23]. Briefly, 1G8 cells were harvested at a density of 3.6–4.0 × 10^6 ^cells/ml and were electroporated at 210 V, 1100 μF, and 48Ω. J558L cells were harvested at a density of 0.8–1.0 × 10^6 ^cells/ml and electroporated at 130V, 1100 μF, and 48 Ω. All transfected cells were harvested 36–40 hours after electroporation and assessed for expression of the reporter genes.

### Luciferase Reporter Assay

Luciferase activity was measured using the Dual-luciferase reporter assay system (Promega) and a TD-20/20 luminometer (Turner Designs, Sunnyvale, CA). Transfection activity was normalized to the activity of the co-transfected *Renilla *luciferase, and the values were calculated as mean ± standard deviation. Statistical analysis was performed using the Student's T-test assuming unequal variances.

### CAT Assay

Cells were lysed in 1× Reporter Lysis Buffer (Promega) and split into two 100 μl aliquots. One of the aliquots was used in the luciferase reporter assay as described above. The second aliquot was heated at 60°C for 10 min. After heating and centrifugation (16,100 × g for 2 min) the supernatant was transferred into the CAT reaction mixture containing ^14^C chloramphenicol, n-Butyryl CoA (Promega) and water to a final volume of 125 μL. The reactions were incubated at 37°C for 16 hours and then terminated by the addition of mixed xylenes (Sigma, St. Louis, MO) to extract the modified chloramphenicol. The extracted layer was then added to liquid scintillation fluid and the radioactive product was measured in a liquid scintillation counter. Assay results were normalized using the luciferase assay results and the values were calculated as mean ± standard deviation. Statistical analysis was performed using the Student's T-test assuming unequal variances.

### Recombinant protein and antiserum production

The sequence encoding the C-terminal region of catfish Oct1 was digested from the pBluescript vector (Stratagene) as an *Eco*RV fragment (taking advantage of restriction sites at position 1149–1154 in the Oct1 coding sequence and in the multiple cloning site of the vector) and ligated into the *Sma*I restriction site of the pQE30 bacterial expression vector (Qiagen) that contains an N-terminal (His)_6 _tag. The plasmid was transformed into the *Escherichia coli *M15 strain (Qiagen) and grown at 37°C in LB medium containing a final concentration of 100 μg/ml ampicillin, 25 μg/ml kanamycin, and induced with 2 mM IPTG (Sigma) 5 hours after reaching an OD_600 _of 0.7. The bacteria were harvested and resuspended in 5 ml sonication buffer (1 mM phenyl methyl sulfonyl fluoride (PMSF), 6 M urea, 400 mM NaCl, 50 mM NaH_2_PO_4 _pH 8.0) and lysed by freeze thawing. A 50% TALLON metal affinity resin slurry (BD Biosciences Clontech) equilibrated with sonication buffer was used to purify the His-tagged Oct1 C-terminal peptide. The eluted protein was dialyzed against 50 mM ammonium bicarbonate and subsequently lyophilized. The purified peptide was used to immunize rabbits (Cocalico Biologicals, Reamstown, PA) using primary immunization with complete Freund's adjuvant and boosting using incomplete Freund's adjuvant.

### Electrophoretic Mobility Shift Assays

A probe was created to the consensus octamer motif for use in EMSA by annealing the oligonucleotides below (the octamer motif is underlined):

Forward Primer (G-656) – 5' CAATATGAATATGCAAATTACCT 3'

Reverse Primer (G-567) – 5' CATAGGTAATTTGCATATTCATA 3'

A probe with a scrambled octamer sequence was produced by annealing the oligonucleotides below (the scrambled octamer motif is underlined):

Forward Primer (G-1249) – 5' CAATATGAATACAAAATATACCT 3'

Reverse Primer (G-1250) – 5' CATAGGTATATTTTGTATTCATA 3'

A probe was created to the first octamer motif located within the core enhancer of Eμ3' using the oligonucleotides below (the octamer motif is underlined):

Forward Primer (G-2110) – 5' GCAAAACACTGCATGTAAATAGTCTAAT 3'

Reverse Primer (G-2111) – 5' CATTATTAGACTATTTACATGCAGTGTT 3'

The scrambled probe to the first octamer motif located within the core enhancer of Eμ3' was created using the oligonucleotides below (the scrambled octamer motif is underlined):

Forward Primer (G-2132) – 5' GCAAAACACTGCTATATGAAAGTCTAAT 3'

Reverse Primer (G-2133) – 5' CATTATTAGACTTTCATATAGCAGTGTT 3'

After annealing, the purity of the double-stranded DNA was verified by gel electrophoresis on a 6% non-denaturing polyacrylamide gel (BioRad). The annealed probes were labeled by a radioactive fill-in reaction using the large fragment of DNA polymerase I (Klenow Enzyme, Fisher, Suwanee, GA) and α-^32^P-dATP (New England Nuclear, Boston, MA) and purified through two Microspin G-25 columns (Amersham Pharmacia Biotech). Nuclear extracts were obtained and EMSA was carried out as described by Ross et al [31]. The TNT quick-coupled transcription/translation system (Promega) was used to generate *in vitro *synthesized catfish Oct1 and Oct2 proteins. ^35^S-Methionine (New England Nuclear) labeled *in vitro *transcribed and translated proteins were analyzed by SDS-PAGE gel electrophoresis and autoradiography. Binding reactions included 4 μl of 5× binding buffer (Promega), 6 μL of TNT products, 2 μL of normal rabbit serum (NRS, anti-Oct1 pre-bleed), cold or scrambled competitor (100 times the concentration of the labeled probe), and anti-Oct1 or anti-Oct2. These reactions were incubated at room temperature for 30 minutes prior to the addition of 1 μL of the radiolabeled probe (10^6 ^cpm/μl). After a 30 minute incubation the DNA-protein complexes were analyzed on a 5% non-denaturing polyacrylamide gel, 150V at 4°C with a recirculating cooling system. Gels were washed in 15% methanol, 5% acetic acid for 30 minutes and allowed to dry, before exposure to a Phosphoimager screen and analysis by a Typhoon Variable Mode Imager (Amersham Biosciences) and the ImageQuant software program.

## Authors' contributions

ML assisted in the design of the study, carried out the sequence alignment, phylogenetic analysis, cloning of expression vectors and DNA constructs, upkeep of cell lines, RT-PCR, transfection studies, *in vitro *protein expression and EMSA, Gal4 activation studies and drafted the manuscript. JH assisted in the design of the study, the participated in the sequence alignment, assisted in the cloning of expression vectors, assisted with upkeep of cell lines, and helped draft the manuscript. DR cloned the Oct1 cDNA, assisted with transfection experiments, carried out in vivo EMSA and participated in revising the manuscript. CK assisted with the transfection experiments, produced the Oct1 antibody and participated in revising the manuscript. MW isolated RNA from catfish tissues and upkeep of cell lines and participated in revising the manuscript. NM assisted with the isolation of RNA, upkeep of cell lines and participated in the revising of the manuscript. GW conceived the study, participated in the design and coordination and helped draft the manuscript. All authors have read and approved the final manuscript.

## Figures and Tables

**Table 1:** Lack of Activation from Catfish Oct1.

**Activation Domain**	**Fold Activation**	**SEM**	
No Activation Domain	1.00	0.00	
VP16	15505.00	2348.00	**
Nucleolin	0.63	0.25	
Oct2β N-terminus	3.00	0.80	p = .057
Oct2β C-terminus	333.00	92.00	*
Oct1 POU Domain	0.35	0.46	
Oct1 N-terminus	-0.25	0.33	*
Oct1 C-terminus	2.00	1.21	

Results reported for the activation of Gal4 fusion proteins as fold activation from background (no activation domain). SEM – Standard Error of the Mean calculated for six replicates. Statistical significance was calculated by the Student T test assuming unequal variances. One asterisk indicates a p value of less than .05 and two asterisks indicate a p value of less than .01.
